# Single Cold Atmospheric Plasma Therapy May Improve the Treatment Outcome of Canine Otitis Externa With Secondary Infection

**DOI:** 10.1111/vde.70027

**Published:** 2025-09-10

**Authors:** Ralf S. Mueller, Cosima Bouassiba

**Affiliations:** ^1^ LMU Small Animal Clinic University of Munich Munich Germany; ^2^ Small Animal Practice, Alte Manufaktur Viersen Germany

**Keywords:** dog, ear, therapy

## Abstract

**Background:**

Otitis externa (OE) with secondary bacterial or yeast infection is a common problem in small animal practice. Cold physical plasma (CPP) has been reported to have antimicrobial activity in vitro.

**Hypothesis/Objectives:**

This randomised, blinded, prospective study assessed the influence of additional CPP treatment on the recovery of canine OE with secondary infection.

**Animals:**

Twenty‐one dogs with bilateral clinical OE and cytological evidence of infection were included.

**Materials and Methods:**

An ear flush was performed with saline solution in all dogs. Additionally, one ear of each dog, chosen in a prospective randomised fashion, was treated with CPP for 30 s. Afterwards, both ears were treated with a depot ear medication containing betamethasone, terbinafine and florfenicol. Seven days later, the depot medication was administered again as recommended by the manufacturer. On Day (D) 0, D7 and D21, a clinical otic score and a validated semiquantitative cytological score were compared with a Friedman test and Dunn's multiple comparison test.

**Results:**

Ears treated additionally with CPP showed lower otic scores after 21 days compared to nontreated ears, although this difference was not significant (*p* = 0.08). Cytological scores improved over 21 days with cocci in CPP‐treated (*p* = 0.003) and nontreated ears (*p* = 0.02). With yeast, there was significant improvement in CPP‐treated (*p* = 0.0002) ears in contrast to nontreated ears. With rods, the improvement was not statistically significant in either group.

**Conclusions and Clinical Relevance:**

CPP treatment seems to be a promising option as an additional treatment after ear flushing.

## Graphical abstract

**Background:** Otitis externa with secondary bacterial or yeast infection is a common problem in small animal practice. Cold physical plasma (CPP) has been reported to have antimicrobial activity in vitro. **Results:** Ears treated additionally with CPP showed lower otic scores after 21 days compared to nontreated ears, although this difference was not significant (*p* = 0.08). Cytological scores improved over 21 days with cocci in CPP‐treated (*p* = 0.003) and nontreated ears (*p* = 0.02). With yeast, there was significant improvement in CPP‐treated (*p* = 0.0002) ears in contrast to nontreated ears. With rods, the improvement was not statistically significant in either group. **Conclusions and Clinical Relevance:** CPP treatment seems to be a promising option as an additional treatment after ear flushing.

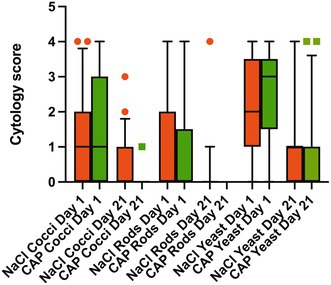

## ZUSAMMENFASSUNG

**Hintergrund:**

Die Otitis externa mit sekundärer bakterieller oder Hefepilz Infektion stellt ein häufiges Problem in der Kleintierpraxis dar. Es wurde beschrieben, dass Kaltplasma (CPP) in vitro eine antimikrobielle Wirkung aufweist.

**Hypothese/Ziele:**

Diese randomisierte, geblindete, prospektive Studie beurteilte den Einfluss zusätzlicher CPP‐Behandlung auf die Verbesserung caniner Otitis externa (OE) mit Sekundärinfektion.

**Tiere:**

Einundzwanzig Hunde mit einer bilateralen klinischen OE und zytologischem Nachweis wurden inkludiert.

**Materialien und Methoden:**

Bei allen Hunden wurde eine Ohrspülung mit Kochsalzlösung durchgeführt. Zusätzlich wurde jeweils ein Ohr eines jeden Hundes, welches in einer prospektiven zufälligen Methode ausgewählt wurde, 30 ss lang mit CPP behandelt. Danach wurden beide Ohren mit einer Depot Ohrmedikation, welche Betamethason, Terbinafin und Florfenicol enthielt, behandelt. Sieben Tage später wurde die Depotmedikation wie vom Hersteller empfohlen nochmals verabreicht. Am Tag (D)0, D7 und D21 wurde ein klinischer Ohrbefund und ein validierter semiquantitativer Zytologiebefund mittels Friedman Test und Dunn´s multiplem Vergleichstest verglichen.

**Ergebnisse:**

Ohren, die zusätzlich mit CPP behandelt worden waren, zeigten nach 21 Tagen niedrigere Ohrwerte im Vergleich zu den unbehandelten Ohren, obwohl dieser Unterschied nicht signifikant (*p* = 0,08) war. Die Zytologie‐Befunde verbesserten sich im Verlauf von 21 Tagen bei den Kokken bei CPP‐behandelten (*p* = 0,003) und nichtbehandelten Ohren (*p* = 0,02). Bei den Hefepilzen gab es signifikante Verbesserungen bei CPP‐behandelten (*p* = 0,0002) Ohren im Gegensatz zu nichtbehandelten Ohren. Bei Stäbchen war die Verbesserung in keiner der Gruppen signifikant.

**Schlussfolgerungen und klinische Bedeutung:**

Die Behandlung mit CPP scheint eine vielversprechende Möglichkeit der zusätzlichen Behandlung nach Ohrspülungen zu sein.

## 摘要

**背景:**

伴随继发性细菌或酵母菌感染的外耳炎在小动物临床中十分常见。据报道，冷物理等离子体(CPP)在体外具有抗菌活性。

**假设/目的:**

本随机、盲法、前瞻性研究评估了在继发感染犬外耳炎(OE)治疗中，额外联合 CPP 治疗对恢复情况的影响。

**动物:**

共纳入 21 只双侧临床 OE 且细胞学证实感染的犬。

**材料与方法:**

所有犬均用生理盐水冲洗耳道。此外，每只犬随机选择一侧耳朵接受 30 秒 CPP 治疗。随后，双耳均使用含有倍他米松、特比萘芬和氟苯尼考的长效耳用药物。7 天后，按生产厂家建议再次使用该长效药物。在第 0 天(D0)、第 7 天(D7)和第 21 天(D21)，采用临床耳评分和经验证的半定量细胞学评分进行比较，并使用 Friedman 检验和 Dunn 多重比较检验。

**结果:**

接受额外 CPP 治疗的耳朵在 21 天后临床耳评分较未接受 CPP 治疗的耳朵更低，但差异无统计学意义(*p* = 0.08)。细胞学评分在 21 天内均有所改善:对于球菌，CPP 治疗耳(*p* = 0.003)和未治疗耳(*p* = 0.02)均显著改善；对于酵母菌，CPP 治疗耳显著改善(*p* = 0.0002)，而未治疗耳无显著改善；对于杆菌，两组均无统计学显著改善。

**结论与临床相关性:**

在耳道冲洗后，CPP 治疗似乎是一种有前景的辅助治疗选择。

## Résumé

**Contexte:**

L’otite externe avec infection bactérienne ou fongique secondaire est un problème courant chez les petits animaux. Il a été rapporté que le plasma physique froid (CPP) avait une activité antimicrobienne in vitro.

**Hypothèse/Objectifs:**

Cette étude prospective randomisée en aveugle a évalué l’influence d’un traitement supplémentaire par CPP sur la guérison de l’otite externe canine (OE) avec infection secondaire.

**Animaux:**

Vingt‐et‐un chiens présentant une OE clinique bilatérale et des signes cytologiques d’infection ont été inclus.

**Matériel et méthodes:**

Un lavage de l’oreille a été effectué avec une solution saline chez tous les chiens. De plus, une oreille de chaque chien, choisie de manière prospective et randomisée, a été traitée avec du CPP pendant 30 secondes. Ensuite, les deux oreilles ont été traitées avec un médicament auriculaire à effet retard contenant de la bétaméthasone, de la terbinafine et du florfenicol. Sept jours plus tard, le médicament à effet retard a été administré à nouveau, conformément aux recommandations du fabricant. Les jours (J) 0, J 7 et J 21, un score clinique auriculaire et un score cytologique semi‐quantitatif validé ont été comparés à l’aide d’un test de Friedman et d’un test de comparaison multiple de Dunn.

**Résultats:**

Les oreilles traitées en plus avec du CPP ont présenté des scores auriculaires plus faibles après 21 jours par rapport aux oreilles non traitées, bien que cette différence ne soit pas significative (*p* = 0,08). Les scores cytologiques se sont améliorés au cours des 21 jours avec des cocci dans les oreilles traitées au CPP (*p* = 0,003) et non traitées (*p* = 0,02). En ce qui concerne les levures, une amélioration significative a été observée dans les oreilles traitées au CPP (*p* = 0,0002) par rapport aux oreilles non traitées. En ce qui concerne les bacilles, l’amélioration n’était statistiquement significative dans aucun des deux groupes.

**Conclusions et pertinence clinique:**

Le traitement au CPP semble être une option prometteuse en tant que traitement complémentaire après un lavage des oreilles.

## 要約

**背景:**

二次的な細菌および酵母菌感染を伴う外耳炎は小動物臨床において一般的な問題である。低音物理プラズマ(CPP)はin vitroにおいて抗菌活性を示すことが報告されている。

**仮説/目的:**

本研究の目的は、本無作為化盲検前向き研究において二次感染を伴う犬の外耳炎(OE)の回復に対する追加CPP療法の影響を評価することであった。

**供試動物:**

臨床的に両側性OEを有し、感染の細胞学的所見を有した21頭を包含した。

**材料および方法:**

全ての犬に生理食塩水による耳洗浄を実施した。追加で前向きランダム方式にて各犬の片方の耳に30秒間CPP治療を施した。その後、両耳にベタメタゾン、テルビナフィンおよびフロルフェニコール含有持続性耳製剤で治療した。7日後に製造元の推奨に基づき持続性薬剤を再投与を施した。D0日、D7日、D21日に、臨床耳スコアおよび検証済み半定量的細胞学的スコアをフリードマン検定およびダン多重比較検定で比較した。

**結果:**

CPPを追加投与した耳は、21日後に非投与耳と比較して耳スコアが低かったが、この差は有意ではなかった(*p* = 0.08)。CPP投与耳(*p* = 0.003)および非投与耳(*p* = 0.02)において、球菌による細胞学的スコアは21日間で改善した。酵母菌は、CPP処理耳(*p* = 0.0002)において未処理耳と対照的に有意な改善が認められた。桿菌では、いずれの群においても改善は統計学的に有意ではなかった。

**結論と臨床的意義:**

CPP治療は、耳洗浄後の追加治療として有望な選択肢と考えられた。

## Resumo

**Contexto:**

A otite externa com infecção bacteriana ou fúngica secundária é um problema comum na clínica de pequenos animais. O plasma físico frio (PFC) demonstrou ter atividade antimicrobiana in vitro*.*

**Hipótese/Objetivos:**

Este estudo prospectivo, randomizado e cego avaliou a influência do tratamento adicional com PFC na recuperação da otite externa canina (OE) com infecção secundária.

**Animais:**

Vinte e um cães com OE clínica bilateral e evidência citológica de infecção foram incluídos.

**Materiais e Métodos:**

Realizou‐se lavagem auricular com solução salina em todos os cães. Além disso, uma orelha de cada cão, escolhida de forma prospectiva e randomizada, foi tratada com PFC por 30 segundos. Posteriormente, ambas as orelhas foram tratadas com uma medicação auricular de depósito contendo betametasona, terbinafina e florfenicol. Sete dias depois, a medicação de depósito foi administrada novamente, conforme recomendado pelo fabricante. Nos dias (D)0, D7 e D21, um escore ótico clínico e um escore citológico semiquantitativo validado foram comparados com o teste de Friedman e o teste de comparação múltipla de Dunn.

**Resultados:**

As orelhas tratadas adicionalmente com CPP apresentaram escores óticos menores após 21 dias em comparação com as orelhas não tratadas, embora essa diferença não tenha sido significativa (*p* = 0,08). Os escores citológicos melhoraram ao longo de 21 dias com cocos nas orelhas tratadas com CPP (*p* = 0,003) e não tratadas (*p* = 0,02). Em relação às leveduras, houve melhora significativa nas orelhas tratadas com CPP (*p* = 0,0002) em contraste com as orelhas não tratadas. Já para bastonetes, a melhora não foi estatisticamente significativa em nenhum dos grupos.

**Conclusões e Relevância Clínica:**

O tratamento com CPP parece ser uma opção promissora como tratamento adicional após a lavagem auricular.

## RESUMEN

**Introduccion:**

La otitis externa con infección secundaria bacteriana o por levaduras es un problema común en la clínica de pequeños animales. Se ha descrito la actividad antimicrobiana del plasma físico frío (CPP) in vitro.

**Hipótesis/Objetivos:**

Este estudio prospectivo, al azar y ciego evaluó la influencia del tratamiento adicional con CPP en la recuperación de la otitis externa canina (OE) con infección secundaria.

**Animales:**

Se incluyeron veintiún perros con OE clínica bilateral y evidencia citológica de infección.

**Materiales y métodos:**

Se realizó un lavado ótico con solución salina en todos los perros. Además, se trató un oído de cada perro, seleccionado de forma prospectiva y aleatoria, con CPP durante 30 segundos. Posteriormente, ambos oídos se trataron con un medicamento ótico de depósito que contenía betametasona, terbinafina y florfenicol. Siete días después, se administró de nuevo el medicamento de depósito según las recomendaciones del fabricante. En los días (D)0, D7 y D21, se compararon la puntuación ótica clínica y la puntuación citológica semicuantitativa validada mediante la prueba de Friedman y la prueba de comparación múltiple de Dunn.

**Resultados:**

Los oídos tratados adicionalmente con CPP mostraron puntuaciones óticas más bajas después de 21 días en comparación con los oídos no tratados, aunque esta diferencia no fue significativa (*p* = 0,08). Las puntuaciones citológicas mejoraron a lo largo de 21 días con presencia de cocos en los oídos tratados con CPP (*p* = 0,003) y no tratados (*p* = 0,02). Con levaduras, se observó una mejoría significativa en los oídos tratados con CPP (*p* = 0,0002) en comparación con los oídos no tratados. Con presencia de bacilos, la mejoría no fue estadísticamente significativa en ninguno de los grupos.

**Conclusiones y relevancia clínica:**

El tratamiento con CPP parece ser una opción prometedora como tratamiento adicional tras el lavado de oídos.

## 1 Introduction

Canine otitis externa (OE) is a common presenting complaint in small animal practice with an annual prevalence of > 7% [1]. Certain breeds such as beagles, basset hounds, golden retrievers and Labradoodles are predisposed [1]. OE is a common feature of canine atopic dermatitis [2, 3, 4], a chronic skin disease predisposing to inflammation and infections of the ear canal. Ear flushing under general anaesthesia using a video‐otoscope is a procedure that may be useful in dogs with chronic OE and otitis media (OM), and can be used to remove microorganisms, purulent debris and cerumen from the canal [5, 6]. It allows evaluation of the ear canal and tympanum, and subsequent treatment is facilitated. Treatment of chronic ear infections may be complicated by the development of biofilm by and antimicrobial resistance in bacteria as well as yeast [7, 8, 9].

Plasma is called the fourth state of aggregation [10]. Based on the plasma source, a distinction is made between thermal and nonthermal plasma sources. Nonthermal or cold physical plasma (CPP) is used in human and veterinary medicine [11]. CPP has a number of effects, particularly on wound healing and infection. Those effects are the result of UV radiation, reactive oxygen and nitrogen species (ROS and RNS), electromagnetic fields and a short‐term increase in temperature associated with CPP treatment. A plasma jet such as the kINPen (kINPen VET; Neoplas GmbH) used in the present study creates a plasma stream, which releases the reactive components, such as ROS and RNS.

In one recent case series, four dogs with OE were treated with CPP in the left and a standard antibiotic/antifungal/corticosteroid combination in the right ear for 2 weeks, and both treatments improved canine OE.

This randomised, controlled, blinded study aimed to determine if a single CPP treatment of canine OE in a larger number of dogs influences the microbial populations in the ear canal directly after use, and the otic and cytological scores in the first 3 weeks after use.

## 2 Material and Methods

### 2.1 Ethics

According to German law, a specific governmental approval is needed for all studies associated with any possible pain or suffering of the dogs included. In this study, flushing of the ears and subsequent treatment with a registered ear medication was medically indicated. The only additional treatment in this study protocol was exposure of one of the ear canals to CPP. CPP is not associated with any pain or discomfort. Additionally, dogs in this study were under general anaesthesia for the ear flush. Consequently, separate approval of this study was not needed under German law.

### 2.2 Inclusion Criteria

Dogs with bilateral OE were included in the study. OE was defined by at least one clinical sign (itching, swelling, tenderness of the external auditory canal, ear discharge, head shaking, redness, papules/crusts and/or exudation) in combination with positive cytological results (≥ 2+ of either cocci, yeasts or rods in both ears based on a validated scoring system for micro‐organisms on cytological evaluation [12]) (see Table 1). All data were recorded on a patient data sheet.

**TABLE 1:** Semiquantitative scoring system for micro‐organisms on cutaneous cytological evaluation [12].

Classification	Description
0	No bacteria/yeast/inflammatory cells
1+	Occasional bacteria/yeast/inflammatory cells present, yet slide must be scanned carefully for detection
2+	Bacteria/yeast/inflammatory cells present in low numbers, yet detectable rapidly without difficulties
3+	Bacteria/yeast/inflammatory cells present in larger numbers and detectable rapidly without any difficulties
4+	Massive amounts of bacteria/yeast/inflammatory cells present and detectable rapidly without difficulties

### 2.3 Exclusion Criteria

Pretreatment with local therapeutic agents and systemic glucocorticoids did not lead to exclusion. However, systemic antibiotics had to have been completed ≥ 48 h before inclusion in the study.

Video‐otoscopy was used to visualise and assess the external auditory canal and the tympanum. Video‐otoscopic and/or radiographic evidence of unilateral or bilateral OM (clouded and/or ruptured tympanum, intact tympanum yet with clear fluid visible through the tympanum or radiographically shadowed bullae tympanicae) led to exclusion from this study. During initial rinsing of the ear with lukewarm physiological saline solution to remove detritus from the external auditory canal, attention was paid to rising air bubbles, which led to the exclusion of the patient from this study as a further sign of OM.

### 2.4 Study Intervention

After inclusion, further interventions were performed under general anaesthesia induced by diazepam 0.5 mg/kg intravenously (Ziapam; Ecuphar), ketamine 5–10 mg/kg iv (Narketan; Vetoquinol) and butorphanol 0.1–0.2 mg/kg iv (Butorgesic; cp‐pharma). If required, propofol (Narcofol; cp‐pharma) was also administered. After intubation, inhalation anaesthesia was conducted with isoflurane (Iso‐Vet; Dechra). Immediately before anaesthesia, meloxicam 0.2 mg/kg sc (Melosolute; cp‐pharma) and metamizole 25–50 mg/kg sc (Vetalgin; MSD) were administered for analgesia.

Physiological saline solution was instilled to clean the external auditory canals and suctioned off with a 5‐mL syringe until no more cerumen/exudate could be mobilised, clear liquid was in the syringe, and the auditory canal was otoscopically free of debris. This procedure was the same for both ears. If necessary, debris that could not be mobilised with saline was carefully removed mechanically from the proximal to the distal ear canal using curettes. The ear flushes took between 10 and 45 min for each ear canal.

**FIGURE 1:**
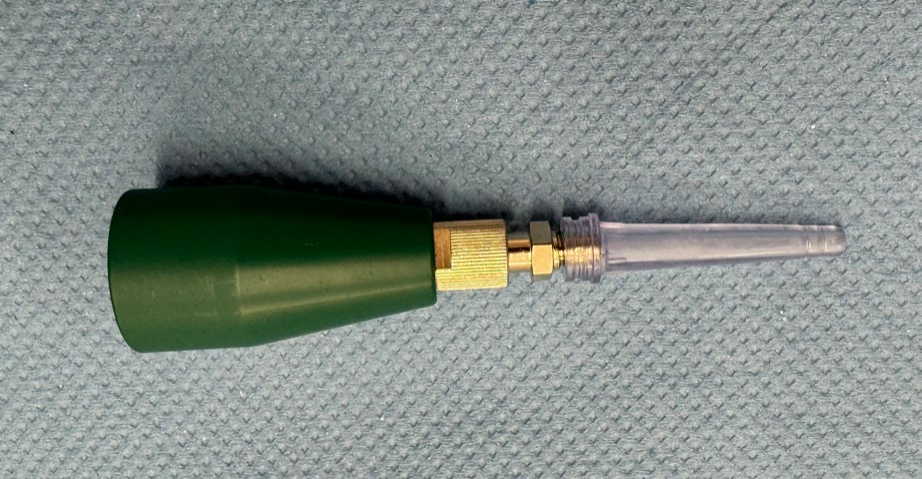
Ear attachment for the kINPen used to fill the ear canal with cold plasma.

On one side, the ears were treated exclusively with isotonic sodium chloride solution as described above, while on the other side they also were treated with CPP. Which side received CPP treatment (kINPen VET) was determined using a computer‐based randomisation function before the study (https://www.graphpad.com/quickcalcs/randMenu/, last accessed 20.1.2025). For the ears treated with CPP, an attachment specifically designed for the ear canal was used. The gas was a mixture of argon as the carrier gas enriched with 1.5% nitrogen and 1.5% oxygen (AIR LIQUIDE Medical). It was applied via a specific otic attachment (Figure 1) that was inserted in the horizontal part of the ear canal and consequently filled up the entire ear canal. The exposure time was 30 s. This application time was chosen based on a study evaluating the exposure time‐dependent genotoxic potential of CPP application [13]. After the application, both ears were rinsed again with isotonic sodium chloride solution of comparable volumes. Subsequently, a trivalent otological agent containing betamethasone acetate, terbinafine and florfenicol was administered in both ears (Osurnia; Elanco).

### 2.5 Bacteriological/Mycological Cultures

Immediately after the ear flush was completed and before the otic medication was administered, a sterile swab was taken from each ear for microbiological examination by rotating the swab by 360° at the transition point from the vertical to the horizontal ear canal. The swabs were marked with the study number and side identification immediately after removal. The swab samples were transported to the laboratory for microbiological examination in Amies medium within 6 h by courier service to an accredited laboratory (Laboklin, Bad Kissingen, Germany) as reported previously [14]. Briefly, swabs were rolled onto a Columbia blood agar (BD Diagnostics) and endoagar (BD Diagnostics) and incubated at 36°C ± 2°C for 18–48 h for bacterial culture. Fungal cultures were performed on Sabouraud chloramphenicol cycloheximide agar (BD Diagnostics) and incubated at 36°C ± 2°C for ≤ 7 days.

Thereafter, the swabs were placed in a Caso enrichment broth (BD Diagnostics) for 18–20 h at 36°C ± 2°C, then the broth was spread on Columbia blood agar and endoagar, and incubated aerobically at 36°C ± 2°C for 18–24 h. Bacterial growth after incubation was evaluated visually, biochemically or with matrix‐assisted laser desorption/ionisation–time‐of‐flight (MALDI‐TOF) (Microflex and Biotyper serius one; Bruker). The testing was carried out according to internationally recognised standardised procedures of the Clinical and Laboratory Standards Institute (CLSI VET08/CLSI M100*). Grown fungi were identified based on their macroscopic and microscopic appearance.

### 2.6 Re‐Evaluations

The first clinical and cytological control took place 7 days later. Directly thereafter, a second application of the trivalent otological agent containing betamethasone acetate, terbinafine and florfenicol (Osurnia; Elanco) was administered according to the manufacturer's instructions.

A similar clinical and cytological re‐evaluation took place 14 days after the first re‐evaluation. Re‐evaluations were always conducted by the same clinician.

### 2.7 Sampling and Staining

Swabs for cytological examination were collected from all ears using a standardised procedure using a sterile otoscope attachment. Swabs were inserted vertically into the canal to the transition point from the vertical to the horizontal ear canal and rotated by 360°. Subsequently, the swabs were rolled out onto glass slides marked with a consecutive study number, air‐dried and heat‐fixed and then stained using a modified Wright's stain (Diff‐Quik; Medion Diagnostics). The stained and air‐dried slides were stored in dedicated containers in a dark, dry place at room temperature until the blinded microscopic evaluation using a validated semiquantitative scoring [14]. The initial swab was evaluated immediately to verify inclusion in the study, yet the slide was subsequently stored until the blinded evaluation.

### 2.8 Clinical Evaluation

In addition to the current study number, the respective date and the clinical classification of the OE for each ear, the individual characteristics of the animal (owner, name, signalment, diseases, medications) as well as the initial status of the disease and the condition during follow‐up examinations after 7 and 21 days were recorded on the patient registration form.

The course of otitis was classified according to the 0–3 Otitis Index Score (OTIS3) using the following characteristics: erythema, oedema/swelling, erosion/ulceration and exudate [15]. Each of those characteristics was graded as 0 (not present), 1 (mild), 2 (moderate) or 3 (severe) and the total score calculated by summing the individual scores.

### 2.9 Cytological Examination

During the microscopic examination (DM750; Leica Mikrosystems AG), the respective sample was first examined at lower magnification in order to detect areas that could be evaluated optimally. The entire slide was then examined and scored for cocci, rods, yeasts, keratinocytes, corneocytes, neutrophil granulocytes and macrophages under oil immersion (×1000). The slides were always evaluated by the same person. The cytological assessment and categorisation were semiquantitative as reported previously [12].

### 2.10 Statistical Methods

In a small pilot study, dogs treated for OE improved to an otic score of 3.0 ± 2.0 after 3 weeks. To detect a further 60% improvement with CPP treatment with a power of 80% and a significance level of 0.05, ≥ 19 dogs were needed. Two more dogs (approximately 10% more) and consequently a total number of 21 dogs were to be included in the study.

The cytological scores and otitis scores before treatment, and 7–21 days after treatment were evaluated for the two therapies using a Friedman test and Dunn's multiple comparison test. The number of ears that were negative for culture (after initial positive cytological results for the respective type of organism—cocci, rods or yeasts) after rinsing was compared using a Fisher exact test. A *p* of < 0.05 was considered significant.

## 3 Results

### 3.1 Study Objects

Twenty‐one dogs with bilateral OE were included in the study.

### 3.2 Clinical Evaluation

**FIGURE 2:**
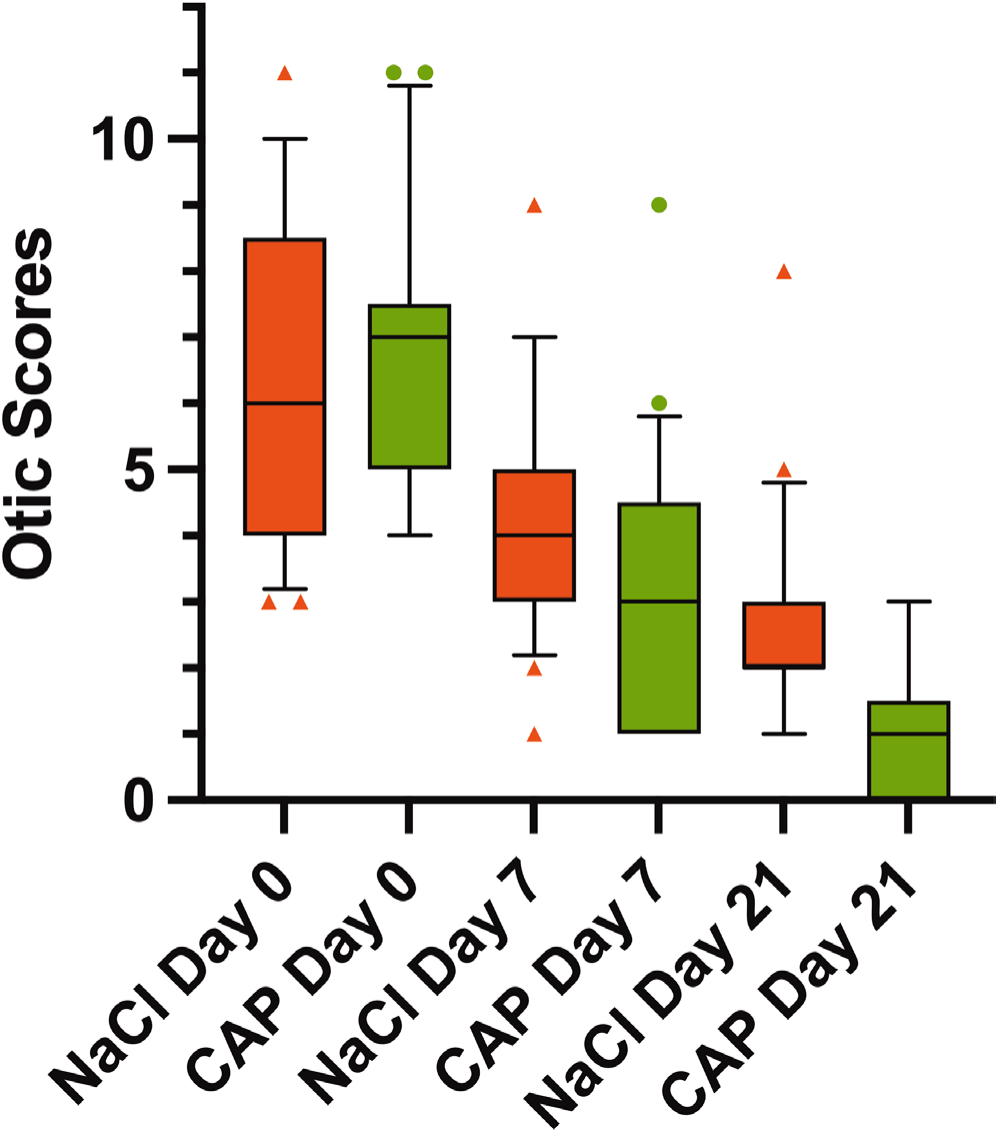
Otic scores of dogs with otitis externa (OE) treated with and without cold physical plasma (CPP) (mean ± standard deviation).

Otic scores improved significantly in all ears between Day (D)1 and D21 (*p* < 0.0001 for the nontreated and for the CPP treated ears). Mean otic scores and standard deviation (SD) are listed in Table 2; all data are listed in Table S1. Ears treated additionally with CPP showed lower otic scores after 21 days compared to nontreated ears, yet the difference did not reach significance (*p* = 0.08; Figure 2).

**TABLE 2:** Otic scores of dogs with otitis treated with and without cold physical plasma (CPP) (mean ± standard deviation [SD]).

	Treatment with CPP	Treatment without CPP
D0	6.7 ± 2.2	6.3 ± 2.5
D7	3.2 ± 2.1	4.4 ± 1.9
D21	1.0 ± 1.0	2.6 ± 1.6

Abbreviation: D, day.

### 3.3 Cytological Evaluation

**FIGURE 3:**
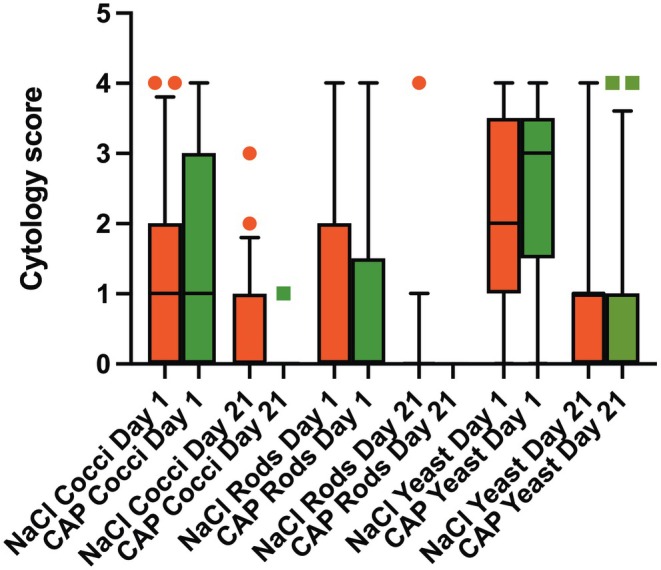
Cytological scores of dogs with otitis treated with and without cold physical plasma (CPP) (mean ± standard deviation).

Cytological scores for cocci improved over 21 days in CPP‐treated (*p* = 0.005) in contrast to nontreated ears (*p* = 0.14), for yeast in CPP‐treated (*p* = 0.001) ears in contrast to nontreated ears (*p* = 0.26). With rods, the numbers decreased, yet the difference did not reach statistical significance (*p* = 0.44 and *p* > 0.99, respectively). Mean cytological scores and their standard deviations for bacterial and yeast organisms are listed in Table 3 (and shown in Figure 3). All data are listed in Table S2.

**TABLE 3:** Cytological scores of dogs with otitis treated with and without cold physical plasma (CPP) (mean ± standard deviation [SD]).

	Treatment with CPP		Treatment without CPP	
	D0	D21	D0	D21
Cocci	1.5 ± 1.6	0.05 ± 0.2	1.5 ± 1.3	0.5 ± 0.8
Rods	0.9 ± 1.5	0 ± 0	0.9 ± 1.5	0.3 ± 0.9
Yeast	2.5 ± 1.4	0.7 ± 1.2	2.2 ± 1.5	1.0 ± 1.4

Abbreviation: D, day.

### 3.4 Evaluation of Bacterial and Fungal Cultures

When evaluating bacterial cultures of dogs with bacterial OE (*n* = 10) after ear flushing without CPP treatment, all cultures were still positive. One of the 10 cultures obtained after treatment with CPP was negative, and this difference was not significant (*p* > 0.999). In CPP‐treated ears of dogs with yeast infections, 6 of 13 cultures did not show any growth. Without CPP treatment, 11 of 18 cultures were negative; that difference also was not significant (*p* = 0.48).

## 4 Discussion

In this randomised, placebo‐controlled, double‐blinded trial treating dogs with bilateral OE, additional CPP treatment for 30 s after ear flushing decreased otic scores significantly at the end of the 21‐day study period.

Cold plasma is generated by supplying energy to a gas to induce partial ionisation. Two main principles are available: dielectric barrier discharges (DBDs) directly generate plasma in atmospheric air onto the treatment target, such as epithelium. A high‐voltage pulse is applied to an electrode covered with an insulating barrier and brought near the target, which acts as the second electrode. By contrast, plasma jets use a second principle and ionise a stream of inert gas that subsequently interacts with oxygen and nitrogen in ambient air. The kINPen uses the latter principle. The bulk of the plasma is created in the device and protrudes from the tip of the device. Plasma was inserted into the ear via a specific otic attachment (Figure 1) into the depth of the horizontal canal, consequently replacing the air in the canal for the duration of the treatment.

Both ears were flushed and subsequently treated in exactly the same way except for a 30 s exposure to CPP on only one side directly after the ear flush. Subsequently, a trivalent otological agent was administered in both ears and repeated after 7 days. This treatment was chosen for several reasons. After the ear flush, it was established that the tympanum was intact and the ear canal was clean, both preconditions for the use of such depot preparations. Additionally, the treatment was administered by the clinician and consequently there was little chance varying owner compliance could influence results. This treatment led to significant improvement of otic scores in both ears after 7 days and even more after 21 days. This is in accordance with another study evaluating this particular preparation [16]. However, there also were lower otic scores in the ears also treated with CPP, compared to those ears that did not receive that treatment, although this difference was not statistically significant.

The administration of a nonsteroidal anti‐inflammatory agent followed by a topical depot preparation containing a glucocorticoid may be viewed critically. However, adverse effects typical for glucocorticoids such as polyuria/polydipsia or polyphagia are not typically seen with this topical preparation, possibly owing to its small amount of systemically active prednisolone. Furthermore, meloxicam was given before the ear cleaning and only lasts a few hours, while the topical glucocorticoid was administered after the procedure and systemic absorption to a relevant degree would need sufficient time. Thus, this combination was not considered an undue risk. None of the dogs showed any adverse effects with the administered treatments.

Antibacterial, antiviral, antifungal and anti‐inflammatory effects, and wound healing properties of the CPP have been known for quite a while and are a subject of intensive research today [10, 17, 18, 19]. No resistance to the effective components of the plasma is known, and there are already promising results in the treatment of multiresistant bacteria in vitro [19]. However, in vivo treatment of wounds postsurgery did not decrease the bacterial burden significantly compared to saline solution [20]. Those contrasting results may be caused by differences in treatment protocols and the inherent differences between laboratory and field studies.

There was no significant difference in the number of negative cultures obtained from the ear canal after ear flushing and (in one of the two ears) exposure of the canal to CPP for 30 s. A recent in vitro study evaluated the bactericidal effects of CPP on *Staphylococcus pseudintermedius* and *Pseudomonas aeruginosa* [17], two important organisms in canine OE [21]. The bactericidal effect of atmospheric plasma against both bacteria increased significantly in a time‐dependent manner, yet complete eradication was only achieved after 90 s of exposure [17]. A similar study reported a minimum of 60 s of exposure for complete eradication of clinical strains of *P. aeruginosa* isolated from canine skin and ear infections [19]. A further study using *Staphylococcus aureus* and *P. aeruginosa* and helium as a working gas also inactivated those bacteria in suspension as well as biofilms after exposure for 120 s [22]. It is possible that the exposure time of 30 s, which was determined at the start of the study according to manufacturer recommendations and before the publication of either of those two studies, was too short to lead to a significant effect. Future studies should use longer exposure times in ears of dogs with OE.

In this study, CPP was used as adjunct treatment in addition to classical ear medications. A recent case series compared treatment of OE exclusively with CPP three times 4 days apart with daily treatment with an ear medication containing miconazole, polymyxin B and prednisolone, and both treatments showed similar results although statistical evaluation was not performed as a consequence of the small case number [23]. The ideal protocol to use CPP in canine OE is not clear and may vary with different underlying diseases and chronicity; thus, further randomised blinded trials are needed. An exposure for 90–120 s may be advisable under anaesthesia after ear flushing. If and how CPP treatment can be used during normal consultations also should be a matter of future clinical research.

There are a number of limitations to this study. The exposure duration discussed above is one of them. Another limitation is the potent depot preparation given in consideration of ethical standards, to standardise treatment and assure absolute compliance, because that led to a significant improvement in the ears not treated with CPP and made it more difficult to detect a difference between groups. Furthermore, although the number of patients needed was calculated in advance based on pilot data, those calculations rely on the pilot study data and a higher standard deviation or a better improvement with medical therapy will then change the power of the study. Higher patient numbers would be desirable in future studies.

In conclusion, CPP seems to be a beneficial treatment option for canine OE based on cytology scores in this study. Whether or not optimised treatment protocols with longer exposure times also can improve microbiological cure rates and otic scores needs to be confirmed with double‐blinded, randomised studies.

## Author Contributions

**Ralf S. Mueller:** conceptualization (lead), formal analysis (lead), methodology (equal), project administration (equal), supervision (lead), validation (lead), visualization (lead), writing – original draft (lead), writing – review and editing (lead). **Cosima Bouassiba:** data curation (lead), investigation (lead), methodology (equal), project administration (equal), resources (lead), validation (supporting), writing – original draft (supporting).

## Conflicts of Interest

Ralf Mueller acted as a consultant or received support for studies or lectures from Artuvet, Bayer Animal Health, Ceva Animal Health, Ecuphar, Elanco Animal Health, Greer Laboratories, Heska Laboratories, Hill's, Royal Canin, MSD Animal Health, Nextmune, Synlab, Virbac Animal Health and Zoetis. Cosima Bouassiba does not report any conflicts of interest.

## Supporting information


**TABLE S1:** Individual otic scores of the 21 dogs with otitis externa included in the study evaluating cold physical plasma (CPP).
**TABLE S2:** Individual cytological scores of the 21 dogs with otitis externa included in the study evaluating cold physical plasma (CPP).

## Data Availability

The data that support the findings of this study are available in the Supporting Information of this article.
