# Supplementary material for: Single Cold Atmospheric Plasma Therapy May Improve the Treatment Outcome of Canine Otitis Externa With Secondary Infection
Source: Vet Dermatol. 2025 Sep 10;37(1):103–10. doi: 10.1111/vde.70027 (PMC12796994; doi:10.1111/vde.70027)
Supplement: Supplementary file 1 — TABLE S1: Individual otic scores of the 21 dogs with otitis externa included in the study evaluating cold physical plasma (CPP). TABLE S2: Individual cytological scores of the 21 dogs with otitis externa included in the study evaluating cold physical plasma (CPP). [file VDE-37-103-s001.pdf]

Supplementary table 1:

NaCl Day 0	NaCl Day 7	NaCl Day 14	CAP Day 0	CAP Day 7	CAP Day 14
4	3	2	4	3	0
6	5	3	6	3	1
4	4	3	4	1	1
5	4	2	4	4	3
6	5	2	7	5	1
9	7	2	9	6	2
6	5	5	7	5	3
4	2	1	7	4	0
8	4	1	11	9	1
3	1	2	5	1	0
3	9	4	5	1	0
8	3	1	8	1	0
10	4	2	6	2	2
10	7	8	7	1	0
4	4	3	5	2	1
9	3	3	11	3	1
11	7	3	10	5	3
7	4	2	7	3	1
5	3	1	7	4	0
7	5	2	6	1	0
4	4	3	4	3	1

Supplementary table 2:

Supplementary table 2a: Cytology scores on D0

NaCl cocci	NaCl rods	NaCl yeast	CAP cocci	CAP rods	CAP yeast
0	0	2	1	0	3
3	0	3	3	0	3
1	0	3	4	0	0
0	0	4	1	0	3
4	0	4	3	0	4
0	0	3	0	0	3
1	0	4	0	0	4
3	0	0	4	0	3
0	1	2	0	4	0
2	4	2	4	4	3
2	0	0	2	4	1
2	4	4	0	2	4
1	2	4	0	0	2
2	4	0	1	0	3
2	0	2	1	0	2
2	0	2	2	0	3
4	0	2	4	0	1
0	2	0	1	1	2
0	2	0	0	3	0
1	0	3	0	0	4
1	0	2	0	0	4

Supplementary table 2b: Cytology scores on D7

NaCl cocci	NaCl rods	NaCl yeast	CAP cocci	CAP rods	CAP yeast
2	0	1	1	0	1
0	0	4	0	0	3
0	0	1	0	0	0
1	0	1	0	0	3
1	0	0	0	0	0
0	0	2	0	0	0
0	0	3	0	0	1
0	0	0	0	0	0
0	0	0	0	4	0
0	0	0	0	0	1
0	4	0	0	0	0
0	0	0	0	0	0
0	0	0	0	0	1
0	4	0	0	0	0
1	0	1	0	0	1
1	0	1	0	0	0
1	0	1	1	0	0
0	0	1	0	0	0
1	0	0	0	0	1
0	0	3	0	0	1
0	0	1	0	0	3

Supplementary table 2c: Cytology scores on D21

NaCl cocci	NaCl rods	NaCl yeast	CAP cocci	CAP rods	CAP yeast
1	0	1	1	0	0
3	0	4	0	0	4
1	0	0	0	0	0
0	0	0	0	0	1
0	0	0	0	0	0
0	0	0	0	0	0
1	0	1	0	0	1
0	0	1	0	0	0
0	0	0	0	0	1
2	0	0	0	0	0
0	0	1	0	0	1
0	0	0	0	0	0
0	0	0	0	0	0
0	4	0	0	0	0
1	0	1	0	0	0
0	0	1	0	0	0
0	0	0	0	0	0
1	1	1	0	0	0
0	0	4	0	0	0
0	0	3	0	0	2
0	1	4	0	0	4
